# Editorial: Natural compounds regulating epigenetics for treating chronic inflammatory diseases

**DOI:** 10.3389/fphar.2022.1121165

**Published:** 2023-01-11

**Authors:** Mingyu Zhang, Ok Joo Sul, Junjiang Fu, Qianqian Wang

**Affiliations:** ^1^ Medical College, Dalian University, Dalian, China; ^2^ University of Ulsan, Ulsan, South Korea; ^3^ Research Center for Preclinical Medicine, Southwest Medical University, Luzhou, China

**Keywords:** natural compounds, traditional Chinese medicine, epigenetic regulation, cancer development, chronic inflammatory diseases

Epigenetics refers to alterations of gene expression without altering DNA sequences, leading to new phenotypes and adding the complexity of the regulation of gene expression (O’Reilly, 2017). Gene expression and cellular function are impacted through the regulation of DNA or histone methylation, acetylation, phosphorylation, ubiquitination modifications and chromatin remodeling by “writer”, “reader” and “eraser” proteins, the whole process being reversible and dynamic (Dawson and Kouzarides, 2012). Recently, post-translational modifications have been deemed as prospective therapeutic strategies for many chronic inflammatory diseases, including but not limited to cancer (Wimalasena et al., 2020), fibrosis (Xue et al, 2021), inflammatory bowel disease (Rajamäki et al., 2021), rheumatic arthritis (Chang et al., 2022), and neurodegenerative diseases (Nikolac Perkovic et al., 2021). Natural compounds are used as bioactive ingredients isolated from natural organisms (plants, fungi, marine organisms, etc.). Most drugs have an “ancestral” structure of a natural compound as a lead molecule, thus allowing the inclusion of natural compounds as very promising drug candidates (Bizzarri et al., 2020). Although substantial progress has been made in the past few years in the discovery of epigenetic drug such as RVX-208 (Wang et al., 2017); in the understanding of targets (Hua et al., 2019) and in the study of compounds in clinical trials, the multiple roles of relevant target enzymes or effector proteins in disease are still unclear, which may be related to novel epigenetic modification mechanisms that have not yet been discovered (Perry et al., 2019). Apart from that, there are significant limitations in the types of chemical structures of existing epigenetic modulators (Fang et al., 2020). These factors have contributed in part to the severe lack of novel epigenetic agonists/antagonists currently available for the treatment of chronic inflammatory diseases. Therefore, there is an urgent need to find novel compounds, especially active small molecules of natural origin, and develop new cell signaling target factors in order to advance the clinical treatment and drug development for specific diseases. Our Research Topic included two reviews and five original research articles on traditional Chinese medicine (TCM), western medicine, and a variety of epigenetic cell signaling pathways.

TCM is a golden resource in China, and is a unique preventive and therapeutic approach that has been used for thousands of years (Ma et al., 2016). Numerous studies on the anticancer benefits of TCM have indicated that it affects DNA methylation changes, in a significant therapeutic manner (Zhu et al., 2022). Five articles on the involvement of herbal medicines in epigenetic regulation for the treatment of related diseases are included in this research theme. Qiu et al. investigated the therapeutic effects of Dabushen decoction (DD) on Osteoarthritis (OA). Through a molecular docking-based virtual screening, they identified five active components of DD (alisol A, emodin, taxifolin, isoliquiritigenin, and schisandrin C) and demonstrated that they could bind stably to DNMT1 and protect PPARγ expression, thereby improving OA. Similarly, Zhang et al. discovered that Lonicerae Japonicae Flos and Forsythiae Fructus herb-pair (LFP) could reduce collagen formation, inflammation, and oxidative stress in the treatment of liver fibrosis. They found that quercetin, luteolin, and kaempferol, which are three components of LFP, not only reduced the symptoms of CCl_4_-induced liver fibrosis but also slowed down inflammation and liver injury. The JAK/STAT signaling pathway is involved in multiple biological processes such as cell proliferation, apoptosis, inflammation, differentiation, immune response, and epigenetics closely related to multiple diseases (Chen et al., 2021). Chen et al. summarized various natural compounds in Chinese herbs that may regulate the JAK/STAT signaling pathway through and the active chemicals isolated from them. This content promotes the insight into the important functions of TCM in regulating STAT signaling pathways and the development of drugs for related diseases. For inflammation treatment, Long et al. found that the anti-inflammatory effects of *Astragalus mongholicu*s polysaccharides (APS) is mediated through the epigenetic modification of m6A in THP-1 macrophages by nephroblastoma 1-associated protein (WTAP). This study displays a fresh idea that APS regulates inflammation at the epigenetic level. In addition, Yang et al., for the first time explored the intervention mechanism of Bai-Mi-Decoction (BMD) with a combined pharmacodynamic and metabolomic approach, verified that it could reduce ischemic stroke (IS) symptoms and brain injury and improve oxidative stress in the mouse model of stroke. This suggests that BMD may be an effective candidate compound for the prevention and treatment of IS. Tian et al. also noted in their investigation of isocitrate dehydrogenase mutant inhibitors that a sterol compound which is an active component of Ganoderma lucidum, had effective inhibitory effects. All these studies can indicate that TCM is a potentially reliable class of epigenetic drugs.

It is worth mentioning that the use of some novel compounds to modulate epigenetic modifications for the treatment of diseases is also an efficient strategy. The mechanism of anti-Alzheimer’s disease (AD) effect of berbamine hydrochloride (BBMH) was investigated by Chen et al. It was found that the anti-AD effect of BBMH may be related to the improvement of microglia and macrophages related functions in mouse brain tissue and the regulation of calcium homeostasis in brain tissue. It was verified that bisbenzylisoquinoline is a multi-targeted anti-AD drug. Moreover, Tian et al. compiled the current status of research on 24 classes of compounds with isocitrate dehydrogenase 1 mutant (mIDH1) as a therapeutic target, the protein that plays an important role as a rate-limiting enzyme in the tricarboxylic acid cycle of cellular metabolic reactions. Recent advances in the development and clinical trials of mIDH1 inhibitors are also provided.

In conclusion, the in-depth study of natural compounds that modulate epigenetics for the treatment of chronic inflammatory diseases is a novel and prospective direction for future drug development and clinical therapy. This editorial highlights recent researchers’ detailed studies on the antagonistic/agonistic effects of epigenetically regulated related proteins from both herbal and western drug routes, providing strong evidence for the important role of small molecule inhibitors in the treatment of multiple chronic diseases. However, the ability to accurately assess the toxicity of epigenetic antagonists/agonists in the organism, how to precisely select key protein targets in diseases, and ideas for structural modifications of natural compounds are questions we need to address in the future.
